# The Teeth and Hair

**Published:** 1894-02

**Authors:** S. H. Guilford

**Affiliations:** Philadelphia.


ARTICLE VI.

### THE TEETH AND HAIR.

BY DR. S. H. GUILFORD, PHILADELPHIA.

Read in Columbian Congress. A synopsis by Mrs. J. M. Walker.

The origin and growth of the hair and teeth are structures most liable to abnormal conditions and are conjointly affected. Evidence of the intimate relations of these tissues, as found in ovarian cysts was considered, and various illustrations of the homology of the two structures given. The conclusions reached were that the intimacy between the two products is probably due to the contemporaneousness of their inception; that when co-ordinately affected the manifestations are variable, though this variability cannot be accounted for in the light of present knowledge.

The ovum of the vertebrates always consists of a mass of protoplasmic matter contained in a connective-tissue envelop. By a process of segmentation the ovum produces many cells that form the blastoderm membrane. This eventually is resolved into three layers, epiblast, mesoblast, and hypoblast. "From the epiblastic or upper layer are formed the epiderm or cuticle of the skin and all its appendages, such as the hair, nails and enamel of the teeth; also the brain and nerves. From the mesoblastic or middle layer are formed the true skin, cartilage, bones, muscles, dentine, cementum of the teeth, etc. From the hyperblastic layer are formed the epithelium of the mucous membrane and the various glands of the alimentary canal." (Cryer.)

We believe all biologists agree that mammalian teeth, if not indeed the teeth of all the vertebrata, are developed in a pouch formed in part and in its earlier stages by a dipping down of the oral epithelium, which in time becomes the enamel. Under this depressed epithelian layer a papilla arises from the corium beneath which the dentine is formed; the papilla being eventually known as the dental pulp. The cementum is formed from what is known as the dental sacculus, a specialized product of the connective tissue which incloses both the enamel and dentinal organs, completely surrounding them.

Such being the origin of the tooth and its different tissues, are we correct in calling the mammalian tooth a "dermal appendage?" The close relationship existing between the teeth of some vertebrates and their dermal scales is best seen in the shark, dog-fish, and other fishes.

Some of our best-known anatomists and physiologists consider the teeth as "specialized dermal appendages," which is accepted by biologists as reasonably conclusive. Each of the various blastodermic layers gives origin to a variety of tissues. Is it, therefore, not reasonable to suppose that where one tissue of a layer is pathologically affected, others of the same layer may be? and if in a number of instances, is it not evident that all of these tissues have the same origin?

In man, we find many evidences of the same relationship between products of the epiblastic layer. In albinism the chief peculiarities are lack of coloring-matter in the skin and hair, and a pink iris. Both of these tissues are epiblastic products, and each is abnormally affected.

Within the past year the writer had the privilege of examining a family of hairless people from the interior of France, consisting of mother, daughter and son. In each individual there was no hair on the scalp, the fine down or lanugo usually covering the body was lacking, and the finger-nails were thick, narrow and pointed, resembling the talons of a bird. The teeth, however, were normal in size, form and number. Wilson mentions the case of a woman, aged thirty-three years, whose entire body was covered with thick and long hair, and who had never perspired. The abnormal growth of hair was not congenial, but began at puberty and remained. The same author speaks of the nails frequently showing evidences of abnormity in connection with either absence or superabundance of hair.

Dr. E. P. Bradbury, of Boston, reports the case of a man, aged twenty-four, who was edentulous and claimed never to have erupted any teeth. This peculiarity was accompanied by an entire absence of saliva. His tongue was dry and leathery, and his speech thick. He had never been able to take solid food of any kind, but subsisted on soups and soft food.

Coming now to the correlation of the hair and teeth, let us consider cases in which an abnormity of one tissue is accomplished by abnormity in the other. In some instances deficiency of one product is accompanied by deficiency in the other, whereas in others deficiency of one is associated with redundancy of the other. Of deficiency of both structures, the most notable instance on record, and perhaps the only one of its character, is that of a man whom the writer exhibited before some medical and dental societies in Philadelphia in 1883.

He was forty-eight years of age, and had never had any teeth, either temporary or permanent. His head was nearly bald, being only slightly covered with fine down; and while hair was present in the public and auxiliary regions, the surface of his body was entirely lacking in the surface-hairs and lanugo usually present. Owing either to the absence or suppression of the sudoriparous glands, he had never perspired. In addition to these peculiarities he had no sense of smell and little of taste. He is still living and in good health, and of his six children only two (girls) show any signs of inherited abnormity, and then only in having about half the usual number of teeth.

There are notable instances of deficiency or abnormity of the teeth or jaws associated with excessive development of the hair.

Julia Pastrana, a Spanish ballet-dancer, had hair on her head dark, coarse and strong, while on her cheeks and chin she wore a full beard several inches long. Her dentition was normal, but the alveolar and overlying soft tissues were so hypertrophied only the coronal surfaces of the teeth could be seen.

The body of Krao, an Indian girl, was exhibited in Europe some years ago, whose body was covered with a well-developed growth of hair, her jaws hypertrophied and her teeth normal. Fedor Jeftichejew had an abnormal growth of hair, associated with a deficiency of teeth. In his lower jaw there are three teeth, one cuspid and two incisors. In the upper jaw there are only two cuspids; one tooth, a lower incisor, having been extracted.

Fedor, now twenty-three years of age, has his entire body covered with a fair growth of hair, while on his face, including the nose and forehead, there is a strong growth of soft hair several inches in length. He perspires but little. The "Burmese Hairy Family" are noted in this country and Europe. Three members of this family, the grandfather, the mother, and Moung Phoset, the son, had their faces and bodies covered with long dark hair of a silky character.

The evidence of the intimate relation of these tissues, is the fact that they are often found together in the ovarian cysts.

"Why should these two products of the epiderm be so often conjointly affected?"

First, the development of the hair and teeth in the human embryo are more nearly contemporaneous than that of the other epidermoid tissues, the dental cap and the first covering of hair or lanugo being both noticeable about the fourth month of fetal life. They are both developed within a sac formed by the dipplng down and unfolding of the epithelium. They are both formed and afterward nourished by a follicle.—*Items of Interest.*

